# Comparative Efficacy and Safety of Antihypertensive Strategies in Diabetic Kidney Disease: Protocol for an Update of a Systematic Review and Component Network Meta‐Analysis

**DOI:** 10.1002/hsr2.73020

**Published:** 2026-08-06

**Authors:** Jamie J. Edwards, Ellesha A. Smith, Yikui Cai, Nicola J. Cooper, Ffion Curtis, Mingyue Deng, Maurice Dungey, Katherine L. Hull, Ananya Nayak, Keith Nockels, James O. Burton, Suetonia C. Palmer, Giovanni Strippoli, Ziwei Wang, Suzanne C. Freeman, Rupert W. Major, Daniel S. March

**Affiliations:** ^1^ Division of Cardiovascular Sciences, School of Medical Sciences University of Leicester Leicester UK; ^2^ Division of Public Health and Epidemiology, School of Medical Sciences University of Leicester Leicester UK; ^3^ Leicester Medical School University of Leicester Leicester UK; ^4^ Liverpool Reviews & Implementation Group (LRiG), Institute of Population Health University of Liverpool Liverpool UK; ^5^ Division of Cancer Sciences, School of Medical Sciences University of Leicester Leicester UK; ^6^ University of Hospitals of Leicester NHS Trust Leicester UK; ^7^ Department of Renal Medicine University Hospitals of Leicester NHS Trust Leicester UK; ^8^ Library and Learning Services University of Leicester Leicester UK; ^9^ Department of Medicine University of Otago Christchurch Christchurch New Zealand; ^10^ Sydney School of Public Health The University of Sydney Sydney New South Wales Australia; ^11^ Department of Precision and Regenerative Medicine and Ionian Area (DiMePre‐J) Nephrology, Dialysis and Transplantation Unit University of Bari Aldo Moro Bari Italy; ^12^ Cochrane Kidney and Transplant, Centre for Kidney Research The Children's Hospital at Westmead Westmead New South Wales Australia

## Abstract

**Background and Aims:**

Chronic kidney disease (CKD) is a significant global contributor to morbidity and mortality, with hypertension and diabetes mellitus remaining its primary causes. Effective antihypertensive treatment is a key modifiable strategy to improve outcomes in this population. However, the emergence of novel therapies, together with uncertainties over the synergistic effects and safety of combining antihypertensive drug classes, has left optimal blood pressure management in CKD and diabetes unclear. We present a study protocol for an updated systematic review and component network meta‐analysis, which aims to answer which blood pressure therapies in adults with diabetes mellitus who have or are at risk of CKD are most efficacious in improving survival and reducing the risk of end‐stage kidney disease.

**Methods:**

This protocol was developed in accordance with the Preferred Reporting Items for Systematic Reviews and Meta‐Analyses Protocols (PRISMA‐P) guidelines and has been registered in the PROSPERO registry (with the identifier: CRD42021286617). Updating a previous review, database searches will be performed in Cochrane Renal Group's specialized register; Embase OVID SP; MEDLINE OVID SP and the Cochrane Central Register of Randomized Trials for randomized controlled trials that evaluate antihypertensive treatments in adults with both CKD and diabetes mellitus. We will include trials that evaluate one or more of the following treatments administered in any combination: (1) angiotensin‐converting enzyme inhibitors; (2) angiotensin receptor blockers; (3) calcium channel inhibitors; (4) beta‐blockers; (5) renin inhibitors; (6) alpha blockers; (7) diuretics; (8) endothelin inhibitors; (9) non‐steroidal and steroidal mineralocorticoid receptor antagonists.

**Results:**

The primary outcomes are all‐cause mortality and progression to end‐stage kidney disease.

**Conclusions:**

This updated systematic review and component network meta‐analysis will incorporate advanced novel analytical techniques to produce the first clinically meaningful hierarchy of antihypertensive interventions according to efficacy and safety in patients with CKD and diabetes mellitus, carrying implications for future clinical practice guidelines.

## 1 Introduction

Chronic kidney disease (CKD) is a major non‐communicable disease contributing significantly to the global burden of morbidity and mortality [1]. An estimated 850 million people worldwide live with CKD, with projections that CKD will progress to the fifth‐leading cause of premature mortality by 2040 [2, 3]. The World Health Organization (WHO) has formally prioritized kidney health with the adoption of a global resolution; this aims to improve international CKD awareness; facilitate earlier detection; enhance CKD prevention; and improve treatment [4].

Hypertension and diabetes mellitus (DM) remain the leading global causes of CKD [5]. Collectively, these three conditions substantially elevate the risk of cardiovascular disease and all‐cause mortality [6]. Among individuals with CKD and DM, effective blood pressure management is one of the most important modifiable strategies for reducing these risks [7]. Antihypertensive medication is prescribed to as many as 60% of people with DM [8]. However, in this population, the pharmacological management of blood pressure has become increasingly complex with the introduction of several new therapeutic agents [9]. Furthermore, the comparative efficacy and safety of available antihypertensive medication classes remain uncertain due to a lack of direct head‐to‐head clinical trials [10, 11]. Certain drug combinations may also pose risks, such as hyperkalemia [9]. Consequently [9], the optimal strategy for blood pressure management in this population remains unclear.

A 2015 network meta‐analysis [9] found no blood pressure‐lowering strategy improved overall survival in adults with DM and CKD, though ACE inhibitors and ARBs were most effective at reducing the risk of ESKD. Its findings have since informed clinical practice guidelines. However, this earlier review employed conventional network meta‐analysis rather than component network meta‐analysis (CNMA), a methodology that models interventions according to their individual treatment components [12]. This approach is particularly relevant in diabetic kidney disease, where patients frequently receive combination antihypertensive therapy, allowing the contribution of individual drug classes to be explored. CNMA may therefore provide additional insights into the relative effects of different drug classes and combinations while making efficient use of the available evidence. By overcoming the lack of head‐to‐head trials, it may also help generate a clinically meaningful hierarchy of treatments in this population.

Given the growing population burden of diabetic kidney disease, the introduction of novel therapies, and advances in analytical methods, we will conduct an updated systematic review and CNMA of the previous Palmer et al. [9] review. This review will investigate the comparative efficacy and safety of antihypertensive strategies in diabetic kidney disease. Ultimately, this review aims to produce the first clinically meaningful hierarchy of antihypertensive interventions according to efficacy and safety in this population.

## 2 Materials and Methods

This systematic review and CNMA were prospectively registered in the PROSPERO registry (with the identifier: CRD42021286617). The protocol of the systematic review and CNMA is reported according to the PRISMA‐P guidelines [13], and the culmination of this review and CNMA will be presented according to the PRISMA 2020 statement (or equivalent update) [14]. The full PRISMA‐P checklist can be found in the supporting information file (Supporting Information S1: Appendix S1). AI was not used to assist with research methodology, data collection or analysis, or literature review processes. This study is a systematic review and component network meta‐analysis of previously published and publicly available data. As such, it does not involve the collection of primary data, human participants, or identifiable personal information. Therefore, ethical approval and informed consent are not required.

### 2.1 Search Strategy

This study will update a previous review using a similar search strategy from that previously published [9] with the novel incorporation of mineralocorticoid receptor antagonist (MRAs) search terms. Electronic databases will be searched for randomized controlled trials (RCTs) published from January 2015 (date following the previous review) and onwards in adults with DM and kidney disease comparing orally administered blood pressure drugs. We will search for all possible comparisons formed by the interventions of interest within the following electronic sources: Cochrane Renal Group's specialized register; Embase OVID SP; MEDLINE OVID SP and the Cochrane Central Register of Randomized Trials (CENTRAL). No restrictions will be applied based on the language of publication. Non‐English reports deemed relevant at the title and abstract screening stage will be included, with final decisions about inclusion made on a case‐by‐case basis at the full‐text screening stage [15].

The full search strategy can be found in the supporting information file (Supporting Information S1: Appendix S2). All identified studies will be imported into covidence systematic review software (Veritas Health Innovation, Melbourne, Australia), where duplicates will be removed, and the screening process will be performed by the research team. The reference lists of systematic reviews identified through the search will also be manually screened separately to identify further relevant studies. The current review process will be merged with an update of the data extraction performed for the previous review [9].

### 2.2 PICO Framework

#### 2.2.1 Population

We will include trials in adults 18 years or older who have type 1 or type 2 DM. We will include participants with DM and evidence of CKD; which will be extracted from three groups of trials: (1) trials that include only participants with CKD and DM; (2) trials with participants who have DM where data for participants with CKD and DM are available; (3) trials of hypertensive participants where data for participants with CKD and DM were available. We will exclude participants who have a functioning kidney transplant or who have end‐stage kidney disease managed with dialysis. Patients with CKD secondary to confirmed causes other than DM will be excluded. Studies that exclusively recruited patients with heart failure will be excluded due to confounding.

### 2.3 Interventions and Comparators

We will include RCTs that evaluate antihypertensive treatments in adults with both CKD and DM. Cross‐over trials will be excluded. Treatments may be administered alone or in combination and must be compared with another treatment, placebo, or usual care. Trials with a follow‐up period of less than 8 weeks for the specified outcomes will also be excluded. We will include trials that evaluate one or more of the following treatments administered in any combination: (1) angiotensin‐converting enzyme (ACE) inhibitors; (2) angiotensin receptor blockers (ARBs); (3) calcium channel blockers; (4) beta‐blockers; (5) renin inhibitors; (6) alpha blockers; (7) diuretics; (8) endothelin inhibitors; and (9) non‐steroidal and steroidal MRAs (labeled Aldosterone Antagonists in previous review). Studies that only included a same‐class comparison (e.g., ARB vs. ARB) were excluded. Follow‐up duration will vary across outcomes; however, primary clinical endpoints (e.g., mortality and ESKD) will be predominantly informed by longer‐term studies.

### 2.4 Outcomes

Table 1 details the relevant primary and secondary outcome data for inclusion.

**Table 1:** Primary and secondary outcomes.

Outcome level	Category	Outcome description
Primary	Efficacy	Total mortality
Primary	Efficacy	End‐stage kidney disease (defined as an estimated glomerular filtration rate below 15 mL/min/1.73 m^2^, treated with long‐term supportive care, dialysis, or transplantation)
Primary	Safety	All serious adverse events (SAEs) as reported in the primary trials
Secondary	Efficacy	Cardiovascular mortality
Secondary	Efficacy	Myocardial infarction (fatal or non‐fatal)
Secondary	Efficacy	Stroke (fatal or non‐fatal)
Secondary	Efficacy	Marker of kidney function (any of: glomerular filtration rate or serum creatinine (continuous measure at end of treatment or change beyond a threshold as defined by the investigators))
Secondary	Efficacy	Blood pressure (any of: systolic blood pressure or diastolic blood pressure at the end of treatment)
Secondary	Efficacy	Proteinuria or albuminuria (as continuous measure (g/24 h or albumin/protein to creatinine ratio) or proportion of patients with albuminuria/proteinuria above author‐specified threshold)
Secondary	Efficacy	Adherence to medication
Secondary	Safety	Dry cough
Secondary	Safety	Presyncope or dizziness
Secondary	Safety	Edema
Secondary	Safety	Falls

### 2.5 Study Screening and Data Extraction

Two reviewers will independently assess all papers for eligibility. Studies will be initially screened by title and abstract, and subsequently by full text if they meet the prespecified inclusion criteria. Any conflicts between reviewers will be resolved by a third reviewer. Before formal screening, all reviewers will complete a calibration exercise by independently screening 10 randomly selected studies to assess inter‐rater agreement and consistency. Conference abstracts and clinical trial records will be assessed during the title and abstract screening stage. Ongoing trials that have not been published will not be included. Records containing sufficient data for pooling will be included; where data are not available, all study authors will be contacted. If the necessary data cannot be obtained, the clinical trial record details will be reported in the supporting materials.

The data will be extracted by two reviewers independently, with a third reviewer assigned to independently process the consensus in data extraction. An initial data extraction form will be piloted and adjusted through Covidence systematic review software based on feedback following the extraction of five sample studies.

We will extract data relating to the study and participants' characteristics, including:

- Setting
- Country (including whether low or middle‐income according to World Bank criteria)
- Baseline blood pressure (continuous variables: systolic and diastolic blood pressure, and proportion of participants who are hypertensive as categorized by the authors)
- Estimated glomerular filtration rate (as calculated and reported by study authors) or creatinine clearance at baseline, or serum creatinine
- Randomized intervention(s) and comparator(s) (including name, class, dose, frequency, route, and duration of administration)
- Non‐randomized or randomized co‐interventions (administered to all treatment groups)
- Primary endpoint
- Trial registration data
- Publication type (letter, journal article, conference proceedings, or unpublished)
- Pro‐equality diversity and inclusion (PRO‐EDI) checklist [16] characteristics.

In addition to this data extraction process, we will use the relevant data extracted from the previously published review [9]. Before analysis, the two extraction templates will be systematically merged to ensure an accurate and comprehensive statistical synthesis that incorporates both review's sets of studies.

### 2.6 Study Quality Assessment

Study quality will be assessed in all included articles using the National Heart, Lung, and Blood Institute (NHLBI) Quality Assessment of Controlled Intervention Studies' tool [17]. This tool includes 14 domains covering key trial design elements, including randomization, allocation concealment, baseline group similarity, application of intention‐to‐treat analysis, blinding procedures, and participants lost in follow‐up. All reviewers will complete a tool calibration and familiarization exercise to assess inter‐rater agreement and accuracy prior to the final study quality assessment. All studies will be assessed for quality by two independent reviewers, with a third reviewer involved to achieve consensus if necessary [18]. We will not quality assess conference abstracts.

### 2.7 Statistical Analysis

All statistical analyses will be performed in R version 4.5.1 [19, 20]. Bayesian modeling will be performed in WinBUGS version 1.4.3 [21] using the R2WinBUGS package [22, 23]. We will conduct frequentist pairwise meta‐analyses by synthesizing trials that compared interventions head‐to‐head using a random‐effects model to incorporate the assumption that different trials assessed different yet related treatment effects [24]. Frequentist pairwise meta‐analysis models will be fitted using the metafor package [25] with heterogeneity assessed using the I^2 statistic [26]. I^2 values over 50% are typically considered substantial [27]. Publication bias will be assessed for the primary outcomes visually through funnel plots.

For dichotomous outcomes, results will be expressed as odds ratios and 95% confidence or credible intervals. For continuous outcomes, results will be expressed as mean difference and 95% confidence or credible intervals when studies report the outcome on the same scale. If different scales have been used, the standardized mean difference or ratio of means will be presented with 95% confidence or credible intervals.

We will conduct a Bayesian component network meta‐analysis (CNMA), with adjustment for multi‐arm studies, to enable the most clinically relevant estimation of the effectiveness of medication classes when used individually or in combination. Adapting the methodology of Welton et al. [12] and Freeman et al. [28], where data allows, we will fit three models: (1) Additive effects model: In this model, we assume each component has a separate effect and that there are no interactions between components so that the total effect of a combination of components is equal to the sum of the individual components. (2) Pairwise interactions model: In this model, pairs of components are allowed to have bigger or smaller effects than the sum of their individual parts, so that the total effect of a combination of components is equal to the sum of the individual components and any pairwise interactions. The combinations of classes selected in the pairwise interactions model will be based on current clinical guidelines for blood pressure management in CKD and DM [10, 29, 30, 31, 32]. Where possible, combinations will consist of any first‐line therapy (ACE inhibitor, ARB, calcium channel blockers, or diuretics) plus any second‐line therapy (alpha blockers, betablockers, or MRAs). (3) Standard NMA model: In this model, every possible combination of components present within at least one study is considered to be a distinct intervention with its own estimate of effectiveness.

For the primary outcomes, we will conduct subgroup analyses limited to studies that evaluate treatment in the following contexts: type 1 DM only, type 2 DM only, microalbuminuria, macroalbuminuria, hypertensive participants, studies with adequate allocation concealment, studies with follow‐up exceeding 24 months, non‐steroidal versus steroidal MRAs, and studies that were not terminated early. Trials without adequate allocation concealment will still be included, but we will account for this potential source of bias in both our analyses and discussion.

To inform the modeling process, for each outcome, the data structure and availability of data will be visualized through a network diagram, a CNMA‐UpSet plot, and CNMA heat map [22]

Heterogeneity will be assessed following the approach of Hartmann‐Boyce et al. [33] by considering the 95% credible interval, median between‐trial standard deviation, and the Deviance Information Criterion (DIC) as indicators of heterogeneity and relative model fit. For the primary outcomes, interventions will be ranked for each MCMC iteration. We will then count how many times each intervention was considered to be the 1st, 2nd, … etc., most effective intervention and express these as percentages. In addition, we will also report the Surface Under the Cumulative Ranking line (SUCRA) for each intervention [34].

Bayesian analyses will be run with three different chains with different initial values, each with at least 30,000 iterations, discarding the first 15,000 iterations. Convergence will be checked through trace plots and the R‐hat statistic. Flat priors for the component effects and between‐trial standard deviation will be used. Sensitivity analysis will be conducted for the primary outcomes, excluding studies identified as being at high risk of bias.

Data will be presented as text, tables, forest plots, network plots, and illustrations.

### 2.8 Study Within a Review

Adjacent to the CNMA, the dissemination of this review will be accompanied by an animated video, developed alongside a patient and public involvement and engagement (PPIE) representative, designed as an educational resource for people living with CKD and DM. The final version of this video will be hosted on the National Kidney Federation (NKF) website alongside other “Kidney Health Videos” and shared through NKF and Kidney Care UK social media channels. This process will be documented by an adjacent Study Within a Review (SWAR), which has been deposited on the SWAR Store on the Northern Ireland Hub for Trials Methodology Research website (https://www.qub.ac.uk/sites/TheNorthernIrelandNetworkforTrialsMethodologyResearch/SWATSWARInformation/Repositories/SWARStore/) under the identifier SWAR51 [34].

In addition, the results will be presented by the PPIE representative at the National Kidney Federation Annual Patients Event. Ultimately, these dissemination plans aim to promote the incorporation of the CNMA results into guidelines and subsequent integrated care systems.

## 3 Discussion

This protocol describes an update of a systematic review using CNMA to evaluate antihypertensive strategies for individuals with CKD and DM. The previous review found that no blood pressure–lowering strategy improved overall survival in adults with diabetic kidney disease, although ACE inhibitors and ARBs were most effective at reducing the risk of ESKD. However, those findings, which shaped current clinical practice guidelines, were based on a conventional network meta‐analysis, which does not adequately capture the combined effects of multiple antihypertensive classes commonly used in clinical practice. CNMA allows the independent and additive effects of individual drug components to be estimated, providing a more realistic synthesis of how therapies are used in combination. This update will also evaluate MRAs as a distinct treatment component, offering new comparative insight into their role alongside standard therapies [12].

The need for this work is underpinned by the scale and impact of CKD, which is estimated to affect between 11% and 13% of the global population [35]. DM and hypertension are the most common causes of CKD [5]. These conditions commonly cluster and are synergistic in their impact on developing CKD and the risk of mortality and/or progression to ESKD [36]. Controlling blood pressure and reducing proteinuria are therefore central to managing CKD in people with DM, aiming to slow disease progression and improve outcomes [37], ultimately relieving pressure on national and global resources [10]. In addition to economic and clinical benefits, identifying optimal antihypertensive strategies supports personalized care in line with national and international initiatives towards precision medicine [38]. It may also contribute to reducing polypharmacy, which could improve long‐term adherence and health outcomes in this population [39]. Overall, this work has the potential to strengthen the evidence base for more targeted antihypertensive therapy and to inform future clinical practice guidelines for people with CKD and DM.

## 4 Conclusion

CKD remains a major global health burden, largely driven by hypertension and DM. By applying CNMA within an updated systematic review, this protocol aims to deliver a clinically meaningful hierarchy of antihypertensive interventions according to both efficacy and safety in patients with CKD and DM. The resulting evidence base will help refine treatment selection and has the potential to shape future clinical practice guidelines.

## Author Contributions

**Jamie J. Edwards:** conceptualization, methodology, writing – review and editing, writing – original draft, project administration. **Ellesha A. Smith:** conceptualization, methodology, writing – review and editing, writing – original draft, funding acquisition. **Yikui Cai:** project administration, methodology, writing – review and editing. **Nicola J. Cooper:** methodology, conceptualization, writing – review and editing. **Ffion Curtis:** writing – review and editing, methodology, conceptualization. **Mingyue Deng:** conceptualization, writing – review and editing, project administration, methodology. **Maurice Dungey:** writing – review and editing, conceptualization, methodology. **Katherine L. Hull:** writing – review and editing, conceptualization, methodology, project administration. **Ananya Nayak:** writing – review and editing, project administration, methodology. **Keith Nockels:** writing – review and editing, methodology. **James O. Burton:** writing – review and editing, methodology, conceptualization. **Suetonia C. Palmer:** methodology, conceptualization. **Giovanni Strippoli:** conceptualization, methodology. **Ziwei Wang:** project administration, methodology. **Suzanne C. Freeman:** conceptualization, methodology, writing – review and editing, writing – original draft, funding acquisition, project administration. **Rupert W. Major:** conceptualization, funding acquisition, writing – original draft, writing – review and editing, methodology, project administration. **Daniel S. March:** conceptualization, funding acquisition, writing – original draft, writing – review and editing, project administration.

## Conflicts of Interest

The authors declare no conflicts of interest.

## Transparency Statement

The lead author, Jamie Edwards, affirms that this manuscript is an honest, accurate, and transparent account of the study being reported; that no important aspects of the study have been omitted; and that any discrepancies from the study as planned have been explained.

## Supporting information


Supporting File

## Data Availability

No new data were generated for this study, as this manuscript presents a protocol for a systematic review and component network meta‐analysis.
