# Supplementary material for: Comparative Efficacy and Safety of Antihypertensive Strategies in Diabetic Kidney Disease: Protocol for an Update of a Systematic Review and Component Network Meta‐Analysis
Source: Health Sci Rep. 2026 Aug 6;9(8):e73020. doi: 10.1002/hsr2.73020 (PMC13448137; doi:10.1002/hsr2.73020)
Supplement: Supplementary file 1 [file HSR2-9-e73020-s001.docx]

**Comparative Efficacy and Safety of Antihypertensive Strategies in Diabetic Kidney Disease: Protocol for an Update of a Systematic Review and Component Network Meta-Analysis**

Jamie J. Edwards^1^., Ellesha A. Smith^2^., Yikui Cai^3^., Nicola J. Cooper^2^., Ffion Curtis^4^., Mingyue Deng^1^., Maurice Dungey^5,6^., Katherine L. Hull^1,7^., Ananya Nayak^3^., Keith Nockels^8^., James O. Burton^1,7^., Suetonia C. Palmer^9^., Giovanni Strippoli^10,11,12^., Ziwei Wang^3^., Suzanne C. Freeman^2^., Rupert W. Major^2,7^ & Daniel S. March^1,7^.

^1^Division of Cardiovascular Sciences, School of Medical Sciences, University of Leicester, Leicester, United Kingdom.

^2^Division of Public Health and Epidemiology, School of Medical Sciences, University of Leicester, Leicester, United Kingdom.

^3^Leicester Medical School, University of Leicester, Leicester, United Kingdom.

^4^Liverpool Reviews & Implementation Group (LRiG), University of Liverpool, Institute of Population Health, L69 3GB.

^5^Division of Cancer Sciences, School of Medical Sciences, University of Leicester, Leicester, United Kingdom.

^6^University of Hospitals of Leicester NHS Trust, Leicester, United Kingdom.

^7^Department of Renal Medicine, University Hospitals of Leicester NHS Trust, Leicester United Kingdom.

^8^Library and Learning Services, University of Leicester, Leicester, United Kingdom (until July 2025).

^9^Department of Medicine, University of Otago Christchurch, Christchurch, New Zealand.

^10^Sydney School of Public Health, The University of Sydney, Sydney, Australia.

^11^Department of Precision and Regenerative Medicine and Ionian Area (DiMePre-J) Nephrology, Dialysis and Transplantation Unit, University of Bari Aldo Moro, Bari, Italy.

^12^Cochrane Kidney and Transplant, Centre for Kidney Research, The Children's Hospital at Westmead, Westmead, Australia.

Correspondence to: Dr Daniel S. March, Department of Cardiovascular Science, University of Leicester, Leicester, UK. Email: dsm12@leicester.ac.uk

Cardiovascular Science, University of Leicester, Leicester, UK. Email: dsm12@leicester.ac.uk

**Supporting Information**

**Appendix S1.**

**Table S1.** *PRISMA-P Checklist.* Source: **Moher D et al:** Preferred reporting items for systematic review and meta-analysis protocols (PRISMA-P) 2015 statement. *Systematic Reviews* 2015 **4:1.**

| **Section/topic** | **#** | **Checklist item** | **Information reported** | | **Line number(s)** |
| --- | --- | --- | --- | --- | --- |
|  |  |  | **Yes** | **No** |  |
| **ADMINISTRATIVE INFORMATION** | | | | | |
| **Title** | | | | | |
| Identification | 1a | Identify the report as a protocol of a systematic review | ☐ | ☐ | 2 |
| Update | 1b | If the protocol is for an update of a previous systematic review, identify as such | ☐ | ☐ | 2 |
| **Registration** | 2 | If registered, provide the name of the registry (e.g., PROSPERO) and registration number in the Abstract | ☐ | ☐ | 41 and 113 |
| **Authors** | | | | | |
| Contact | 3a | Provide name, institutional affiliation, and e-mail address of all protocol authors; provide physical mailing address of corresponding author | ☐ | ☐ | 4-24 |
| Contributions | 3b | Describe contributions of protocol authors and identify the guarantor of the review | ☐ | ☐ | 332-335 |
| **Amendments** | 4 | If the protocol represents an amendment of a previously completed or published protocol, identify as such and list changes; otherwise, state plan for documenting important protocol amendments | ☐ | ☐ | 116-268 |
| **Support** | | | | | |
| Sources | 5a | Indicate sources of financial or other support for the review | ☐ | ☐ | 325-330 |
| Sponsor | 5b | Provide name for the review funder and/or sponsor | ☐ | ☐ | 325-330 |
| Role of sponsor/funder | 5c | Describe roles of funder(s), sponsor(s), and/or institution(s), if any, in developing the protocol | ☐ | ☐ | 328-329 |
| **INTRODUCTION** | | | | | |
| **Rationale** | 6 | Describe the rationale for the review in the context of what is already known | ☐ | ☐ | 83-107 |
| **Objectives** | 7 | Provide an explicit statement of the question(s) the review will address with reference to participants, interventions, comparators, and outcomes (PICO) | ☐ | ☐ | 102-107 |
| **METHODS** | | | | | |
| **Eligibility criteria** | 8 | Specify the study characteristics (e.g., PICO, study design, setting, time frame) and report characteristics (e.g., years considered, language, publication status) to be used as criteria for eligibility for the review | ☐ | ☐ | 116-161 |
| **Information sources** | 9 | Describe all intended information sources (e.g., electronic databases, contact with study authors, trial registers, or other grey literature sources) with planned dates of coverage | ☐ | ☐ | 116-131 |
| **Search strategy** | 10 | Present draft of search strategy to be used for at least one electronic database, including planned limits, such that it could be repeated | ☐ | ☐ | Table S1 |
| ***STUDY RECORDS*** | | | | | |
| Data management | 11a | Describe the mechanism(s) that will be used to manage records and data throughout the review | ☐ | ☐ | 128-131 |
| Selection process | 11b | State the process that will be used for selecting studies (e.g., two independent reviewers) through each phase of the review (i.e., screening, eligibility, and inclusion in meta-analysis) | ☐ | ☐ | 162-176 |
| Data collection process | 11c | Describe planned method of extracting data from reports (e.g., piloting forms, done independently, in duplicate), any processes for obtaining and confirming data from investigators | ☐ | ☐ | 162-176 |
| **Data items** | 12 | List and define all variables for which data will be sought (e.g., PICO items, funding sources), any pre-planned data assumptions and simplifications | ☐ | ☐ | 177-191 |
| **Outcomes and prioritization** | 13 | List and define all outcomes for which data will be sought, including prioritization of main and additional outcomes, with rationale | ☐ | ☐ | Table 1 |
| **Risk of bias in individual studies** | 14 | Describe anticipated methods for assessing risk of bias of individual studies, including whether this will be done at the outcome or study level, or both; state how this information will be used in data synthesis | ☐ | ☐ | 197-206 |
| ***DATA*** | | | | | |
| **Synthesis** | 15a | Describe criteria under which study data will be quantitatively synthesized | ☐ | ☐ | 208-220 |
|  | 15b | If data are appropriate for quantitative synthesis, describe planned summary measures, methods of handling data, and methods of combining data from studies, including any planned exploration of consistency (e.g., *I* ^2^, Kendall’s tau) | ☐ | ☐ | 221-259 |
|  | 15c | Describe any proposed additional analyses (e.g., sensitivity or subgroup analyses, meta-regression) | ☐ | ☐ | 238-244 |
|  | 15d | If quantitative synthesis is not appropriate, describe the type of summary planned | ☐ | ☐ |  |
| **Meta-bias(es)** | 16 | Specify any planned assessment of meta-bias(es) (e.g., publication bias across studies, selective reporting within studies) | ☐ | ☐ | 208-215 |
| **Confidence in cumulative evidence** | 17 | Describe how the strength of the body of evidence will be assessed (e.g., GRADE) | ☐ | ☐ |  |

**Appendix S2**

***Table S2.*** *Full Search Strategy (Source: [9]*).
